# Bluetongue Virus VP1 Polymerase Activity *In Vitro*: Template Dependency, Dinucleotide Priming and Cap Dependency

**DOI:** 10.1371/journal.pone.0027702

**Published:** 2011-11-15

**Authors:** Eiko Matsuo, Polly Roy

**Affiliations:** Faculty of Infectious and Tropical Diseases, London School of Hygiene and Tropical Medicine, London, United Kingdom; Chungbuk National University, Republic of Korea

## Abstract

**Background:**

Bluetongue virus (BTV) protein, VP1, is known to possess an intrinsic polymerase function, unlike rotavirus VP1, which requires the capsid protein VP2 for its catalytic activity. However, compared with the polymerases of other members of the *Reoviridae* family, BTV VP1 has not been characterized in detail.

**Methods and Findings:**

Using an *in vitro* polymerase assay system, we demonstrated that BTV VP1 could synthesize the ten dsRNAs simultaneously from BTV core-derived ssRNA templates in a single *in vitro* reaction as well as genomic dsRNA segments from rotavirus core-derived ssRNA templates that possess no sequence similarity with BTV. In contrast, dsRNAs were not synthesized from non-viral ssRNA templates by VP1, unless they were fused with specific BTV sequences. Further, we showed that synthesis of dsRNAs from capped ssRNA templates was significantly higher than that from uncapped ssRNA templates and the addition of dinucleotides enhanced activity as long as the last base of the dinucleotide complemented the 3′ -terminal nucleotide of the ssRNA template.

**Conclusions:**

We showed that the polymerase activity was stimulated by two different factors: cap structure, likely due to allosteric effect, and dinucleotides due to priming. Our results also suggested the possible presence of *cis*-acting elements shared by ssRNAs in the members of family *Reoviridae*.

## Introduction

Viral RNA-dependent RNA polymerases (RdRps) share a similar catalytic mechanism as well as a similar structure, including conserved sequence motifs and catalytic residues [1], [2], [3], [4], [5]. Despite these similarities, each RdRp has different ways to recognize RNA templates, initiate RNA synthesis, elongate the RNA chains and regulate those activities [6], [7], [8], [9]. For segmented, double-strand RNA (dsRNA) viruses, including *Reoviridae* family members, RNA synthesis by RdRp occurs within a capsid and is capable of reading both single- and double-strand RNAs in association with other inner viral proteins (polymerase complex) in the absence of host factors [2], [10], [11], [12], [13], [14], [15], [16], [17]. It is believed that this specific feature of dsRNA viruses allows their RdRps to synthesize ssRNA transcripts (mRNAs) from viral genomic dsRNA segments and viral genomic dsRNAs from ssRNA transcripts without exposing their genomic dsRNA to the host innate immunity sensors [18]. Recently, it was reported that dsRNAs which were detected outside the rotavirus viroplasm seemed to activate PKR [19].

Bluetongue virus (BTV), the etiological agent of Bluetongue disease of livestock, is a member of the Orbivirus genus of the family *Reoviridae*. BTV particles have three consecutive layers of proteins organized into two capsids, an outer capsid of two proteins (VP2 and VP5) and an inner capsid or “core” composed of two major proteins, VP7 and VP3 which encloses the three minor proteins VP1, VP4 and VP6 in addition to the viral genome. The viral genome is segmented and consists of ten linear dsRNA molecules, classified from segment 1 to segment 10 in decreasing order of size (S1-S10) [20], [21]. After cell entry, the outer capsid is removed to release a transcriptionally active core particle, which provides a compartment within which the ten genome segments can be repeatedly transcribed by core-associated enzymes including VP1, VP4 and VP6 [14], [15]. Ten mRNAs are synthesized from the ten genome segments and released from the core particle into the host cell cytoplasm where they act as templates both for translation and for negative strand viral RNA synthesis to generate genomic dsRNAs [14], [22]. Previously, we demonstrated that purified BTV VP1 is active as replicase synthesizing dsRNA from positive strand ssRNA *in vitro* in the absence of any other viral protein [23], [24]. However, the catalytic activity of VP1 was not further characterized.

The crystal structures of RdRp proteins have been reported for two members of the family, λ3 of reovirus and VP1 of rotavirus [3], [4]. Both RdRp showed a similar cage-like structure with four well-defined tunnels that allow access of the template RNAs, nucleotides and divalent cations to the internal catalytic site, as well as two distinct exit channels for template RNA and products [25]. Although the crystal structure of BTV VP1 is not known, a secondary structure-based three-dimensional model of BTV VP1 revealed structural similarity to other *Reoviridae* polymerases [26]. Despite this structural similarity, BTV VP1 exhibits two distinct functional features which distinguish it from rotavirus VP1 [24]. Firstly, BTV VP1 exhibits RNA replicase activity in the absence of all other virus encoded proteins, whereas rotavirus VP1 requires VP2, which forms the inner layer of the virus particle, for its activity [27], [28], [29]. Secondly, our initial study indicated that BTV VP1 does not require the 3′ conserved region for *in vitro* dsRNA synthesis, unlike rotavirus VP1, which recognizes the UGUG tetranucleotide of the 3′ end conserved sequence [4], [24], [29], [30], [31], [32], [33]. Nevertheless, during virus replication all three proteins, BTV VP1, reovirus λ3 and rotavirus VP1, as well as dsRNA bacteriophage phi 6 RdRp, essentially perform the same function [3], [5], [24], [34], [35], [36], [37].

The crystal structure of the reovirus λ3 and rotavirus VP1 also showed that there are ‘cap’ binding sites on the surface of the cage-like structure [3], [4], suggesting that the cap appears to be the primary element by which VP1 docks and recognizes the 5′ end of a plus strand [4], [25]. The activity of influenza virus-associated polymerase, which is well known to have a cap-snatching mechanism, can be stimulated by cap-1 structures (^m7^GpppN^m^) as well as dinucleotides, such as ApG and GpG [38], [39], [40], [41], [42], [43], [44], [45]. The regulation of transcription by cap structures was also reported in *Bunyaviridae* [46]. Unlike dinucleotides, the cap-1 structure functions as an allosteric regulatory factor, rather than by priming transcription, with enhanced RNA synthesis by influenza virus-associated polymerase [40], [43], [44], [45]. Furthermore, the cell-free system for rotavirus RNA polymerase revealed the specific priming of minus strand RNA synthesis by a dinucleotide rather than dinucleoside monophosphate [32], and formation of the initiation complex with dinucleotide and template, unlike the RdRp of dsRNA bacteriophage, phi6, which does not require a primer for initiating dsRNA synthesis but has a “back-priming” initiation mechanism [47], [48]. These previous studies strongly suggest that the cap structure or dinucleotide may have an effect on the polymerase activity of BTV VP1.

In this study, we investigated the factors that affect BTV VP1 *in vitro* catalytic activity including the requirement of RNA sequences that are recognized by BTV VP1, priming and other co-regulating factors. We first confirmed the robustness of polymerase activity by demonstrating that VP1 could synthesize all ten dsRNA segments from ten individual ssRNA segments in a single reaction. Further, we showed that *in vitro* polymerase activity of VP1 is sequence-independent and could synthesize genomic dsRNAs of the other orbiviruses or rotaviruses when provided with ssRNA templates of these heterologous viruses. In contrast, dsRNAs were not synthesized from non-viral ssRNA templates by VP1, unless those were fused with some specific BTV sequences, indicating the presence of *cis*-acting elements shared by members of the family *Reoviridae*. Moreover, our data showed that the activity was enhanced both in the presence of a cap structure or a dinucleotide; although their roles appear to be distinct, one appears to be allosteric while the other is required for priming.

## Results

### Polymerase activity of BTV VP1 is highly efficient *in vitro*

**Figure 1:**
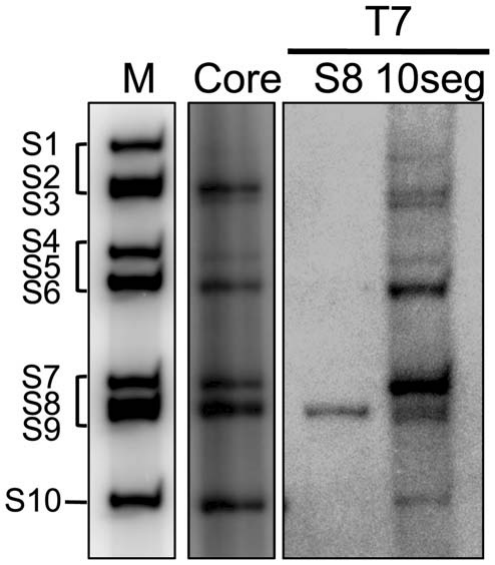
Efficient polymerase activity of BTV VP1 *in vitro.* BTV core-derived ssRNAs (lane 2), single S8 T7 ssRNA (lane 3) and all ten segments T7 ssRNAs (lane 4) were used for the *in vitro* polymerase assay as described in Materials and Methods and synthesized dsRNA were analyzed by PAGE. The radiolabeled dsRNAs were detected by autoradiography using Storage Phospher screen and image analyzer, Typhoon Trio. The end-labeled BTV1 genome segments were used as markers (lane 1). Genome segments are indicated.

BTV VP1 has already been reported to have the ability to synthesize dsRNA from BTV T7 ssRNA templates [24]. However, it has not been previously shown that polymerase proteins of any members of the family *Reoviridae* can synthesize the complete set of genomic dsRNA *in vitro* in a single reaction mixture. Since purified BTV VP1 alone could synthesize a duplex on a single ssRNA template, we attempted to assess if VP1 could synthesize all ten dsRNAs of BTV in a single reaction. Either 1.0 µg of the complete set of *in vitro* synthesized ssRNAs from viral cores (core ssRNAs) or *in vitro* generated ten ssRNAs from T7 plasmids (T7 ssRNAs) [49], [50], each approximately 0.1 µg, were used as templates together with approximately 70 ng of purified VP1, significantly less VP1 than was used previously [24], for *in vitro* polymerase assay. Both reactions were carried out at 37°C for 5 h and dsRNAs were purified from the reaction mixtures. The dsRNA profiles of each reaction, analyzed by 7% native PAGE gel, demonstrated clearly that purified VP1 synthesizes all ten dsRNAs in a single reaction mixture and could utilize efficiently both authentic core ssRNAs and the T7 ssRNAs (Fig. 1). The amount of each synthesized segment was not equal. However, when each single T7 ssRNA template was used for separate reactions, the dsRNA from each T7 ssRNA was synthesized equally well (data not shown) [24].

The data also showed that BTV VP1 could efficiently synthesize all ten dsRNAs in the absence of any other protein, whereas rotavirus VP1 required VP2 for single dsRNA synthesis, suggesting that the recombinant BTV VP1 possesses robust polymerase activity *in vitro*.

### BTV polymerase is capable of synthesizing genomic RNAs of rotavirus and other members of family *Reoviridae*

**Figure 2:**
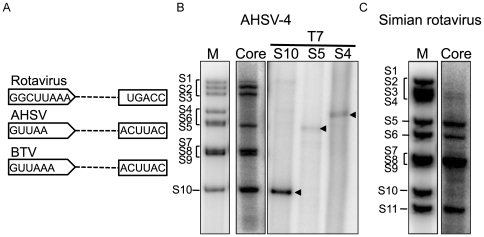
Sequence-independent polymerase activity of BTV VP1. A. Schematic representation of conserved regions at both 5′ and 3′ end of rotavirus, AHSV and BTV. B. AHSV-4 core-derived ssRNAs (lane 2) and AHSV-4 T7 ssRNAs (S10, lane 3; S5, lane 4; S4, lane 5) were used as templates. The radiolabeled dsRNAs were detected by autoradiography. The end-labeled genome segments were used as markers (lane 1). Arrows indicate synthesized dsRNA. Note that AHSV4 S6 migrates slower than AHSV4 S5. C. Simian rotavirus (SA11) DLP ssRNAs (lane 2) were used as templates. The end-labeled genome segments of simian rotavirus (SA11) were used as markers (lane 1). Genome segments are indicated.

The data above confirms that BTV VP1 is not only functional on its own and requires no other viral protein but that the activity is also highly efficient *in vitro*. Thus, it is possible that VP1 may be capable of using other related viral ssRNAs as templates. We selected another member of the Orbivirus genus, African Horse Sickness virus, AHSV, the genomic RNA segments of which have 5′ and 3′ conserved regions similar to those of BTV genomes (Fig. 2A). For this study, we used the complete set of ssRNAs of AHSV-4, synthesized *in vitro* from purified cores, the completeness of which were confirmed by reverse genetics [51]. In parallel, we also used several T7 ssRNAs (S4, S5 and S10) of AHSV-4 as templates for *in vitro* polymerase assay. Polymerase reactions were carried out with either the core ssRNA templates or the single T7 ssRNA templates as described above. When purified dsRNAs were analyzed by 7% native-PAGE gel, dsRNA segments were detectable from each template (Fig. 2B).

**Figure 3:**
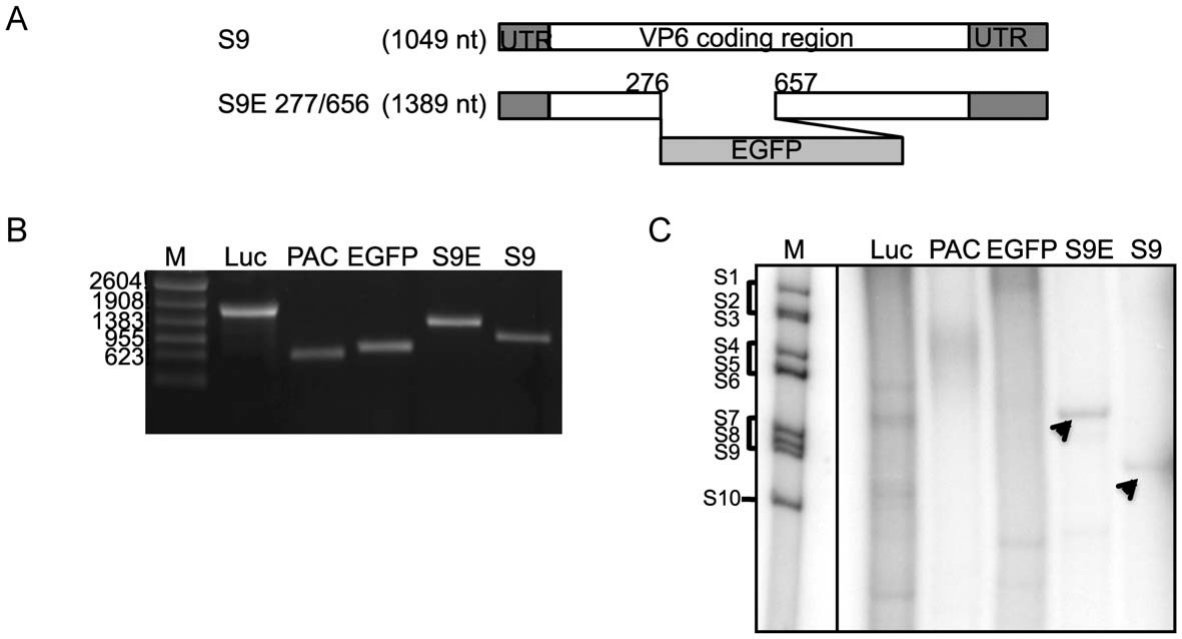
Requirement of *cis*-acting element for BTV polymerase activity. A. Schematic representation of modified S9 transcripts [50]. B. ssRNA templates were synthesized using T7 polymerase. Lane 1, ssRNA markers; lane 2, luciferase gene (about 1800 nucleotides); lane 3, PAC gene (about 609 nucleotides); lane 4, EGFP gene (about 729 nucleotides); lane 5, chimeric S9-EGFP, EGFP277/657, (1389 nucleotides) and lane 6, wild type BTV10 S9 (1049 nucleotides). The numbers on the left indicate the lengths of the markers in nucleotides. C. dsRNA synthesis from luciferase gene (lane 2), PAC gene (lane 3), EGFP gene (lane 4) and chimeric S9-EGFP, EGFP277/657, ssRNA template (lane 5) was compared with wild type BTV10 S9 (lane 6). The radiolabeled bands were detected by autoradiography. The arrows indicate the bands with the correct size. The end-labeled BTV1 genome segments were used as markers (lane 1). Genome segments are indicated on the left.

To investigate further if VP1 could synthesize dsRNA on an ssRNA template of another member of the family, we selected rotavirus ssRNA templates derived from rotavirus double-layered particles (DLPs), which are equivalent to BTV cores, *in vitro*. Rotavirus DLP ssRNAs (11 segments) were generated *in vitro* from purified DLPs of SA11, a strain of simian rotavirus, and again used as templates as described. Surprisingly, although genome sequences of rotaviruses are different from those of BTV serotypes (Fig. 2A), BTV VP1 could indeed synthesize dsRNAs from each rotavirus DLP ssRNAs (Fig. 2C). Similar results were obtained when rhesus rotavirus DLP ssRNAs or bovine rotavirus DLP ssRNAs were used as templates (data not shown). These results confirmed that the *in vitro* polymerase activity of BTV VP1 is sequence-independent at least within the family *Reoviridae*. Alternatively, viruses belonging to the family *Reoviridae* may share some motif or elements in their genomic RNAs, which may be required for polymerase activity. To verify this further, three non-viral ssRNAs, a luciferase gene ssRNA (∼1800 nucleotides), a puromycin resistant gene ssRNA (PAC, 609 nucleotides) and an EGFP gene ssRNA (729 nucleotides) (Fig. 3B) were used as templates. In addition, one chimeric S9-EGFP gene (S9E 277/657) (Fig. 3A and B), which was established previously as a functional segment *in vivo* [50], was also used as a template. None of the non-viral ssRNAs could serve as templates to generate correct duplexes (Fig. 3C, lanes 2–4). However, when the chimeric S9-EGFP gene, in which BTV RNA sequences were fused with EGFP RNA, was used as a template, a “perfect” chimeric dsRNA was synthesized similar to the wild-type S9 (Fig. 3C, lanes 5 and 6). These data suggested that some sequence-based character of BTV segments, such as certain secondary structure of ssRNA, is necessary for polymerase activity of BTV. It is possible that other members of the family may also possess similar characteristics.

### Does Cap structure of ssRNA stimulate polymerase activity?

**Figure 4:**
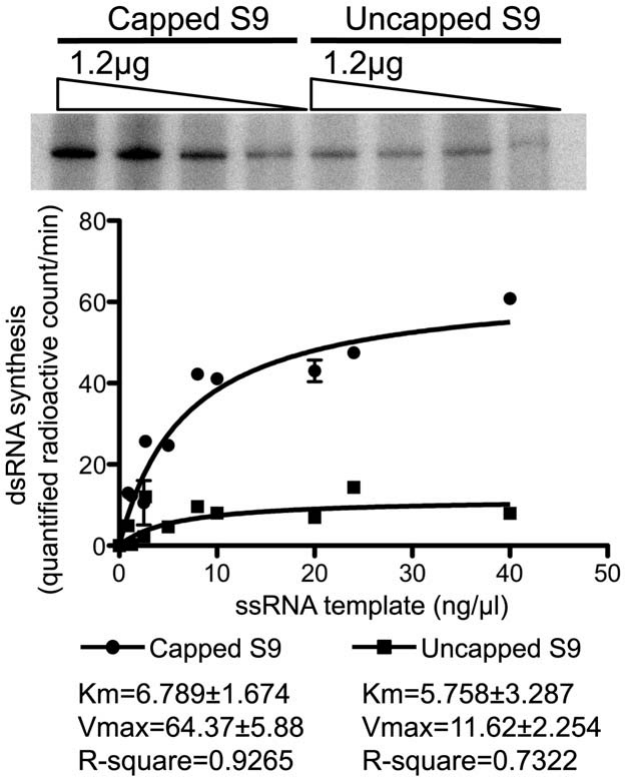
Comparison of the dsRNA synthesis between capped and uncapped T7 S9 ssRNA. Serial diluted (2-fold and 3-fold) capped or uncapped T7 S9 ssRNAs were added to the reaction mixture containing 70 µg of VP1 (upper panel). Several serial dilutions of capped (circle) or uncapped (square) T7 S9 ssRNAs were performed. The radiolabeled bands were detected by autoradiography. The intensity of each band was quantified using ImageJ software (NIH: http://rsb.info.nih.gov/ij/) and plotted on the graph (lower panel). The fitted curves shown on the graph were calculated using the program Prism (GraphPad Software, USA). The kinetics parameters were determined by the Michaelis-Menten equation as described in Materials and Methods and shown on the graph.

It is known that a 5′ cap structure can regulate viral polymerase activity as well as stabilize ssRNA [40], [43], [44], [45], [46]. Additionally, the crystal structure of reovirus λ3 and rotavirus VP1 revealed that it has cap-binding sites on the surface of its cage-like structure, close to the entry channel for template ssRNA [3], [4]. Similarly, the study on rotavirus polymerase VP1 suggests that the cap appears to be the primary element by which VP1 docks and recognizes the 5′ end of a plus strand RNA [4]. Previous work has shown that T7-derived uncapped BTV ssRNAs could serve as templates for *in vitro* polymerase reactions but a role for the cap structure as an enhancing element was not investigated [24]. Since the positive-sense RNAs of the BTV genome possess a cap structure at the 5′ end, we assessed the role of the cap structure on the polymerase activity of BTV VP1. Thus, the efficiency of dsRNA synthesis was compared between capped T7 S9 ssRNA and uncapped T7 S9 ssRNA templates. Several serial dilutions (2-fold and 3-fold) of both types of templates were used for polymerase assay and the final dsRNA products were analyzed by native PAGE gel as described above (Fig. 4, upper panel). The radioactivity of each dsRNA band was quantified by using ImageJ software as described in Materials and Methods (Fig. 4, lower panel). The yield of dsRNAs from capped T7 S9 ssRNAs was higher than that from uncapped T7 S9 ssRNAs (Fig. 4). No significant difference was observed between the Km value of capped T7 S9 ssRNA and that of uncapped T7 S9 ssRNA (6.78±1.674 vs 5.75±3.287, P>0.05), suggesting that the presence of cap structure at 5′ end did not affect on the affinity of ssRNA template to VP1. However, the Vmax value of capped T7 S9 ssRNA was approximately six fold higher than that of uncapped T7 S9 ssRNA (64.37±5.88 vs 11.62±2.254, P<0.01), suggesting that the presence of cap structure at 5′ end of ssRNA template increased VP1 catalytic activity. Moreover, dsRNA synthesis was saturated by approximately 0.5 µg of input ssRNA template, regardless of being capped or not. In addition, although the amount of ssRNA decreased after 5 h of reaction, uncapped ssRNA still remained intact after the reaction (data not shown). These results suggested that the lower efficiency of dsRNA synthesis from uncapped ssRNAs was not due to template instability. These results support a model in which the cap structure of the template influences the catalytic activity of BTV VP1.

**Figure 5:**
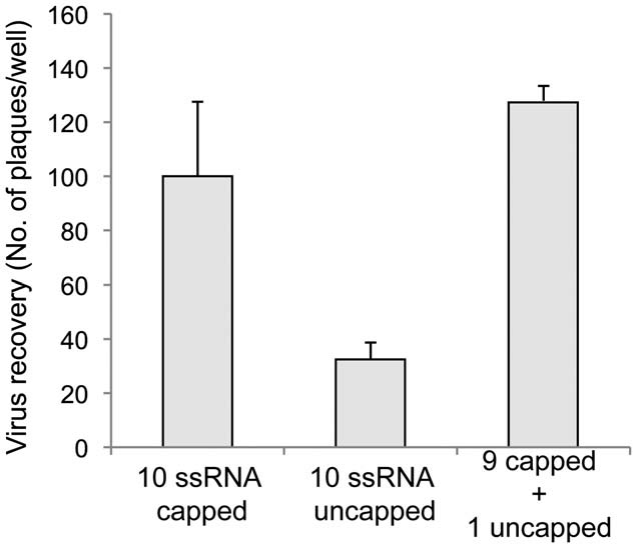
Recovery of BTV from uncapped ssRNA. The recovery of BTV from RNA generated in vitro was compared using uncapped and capped ssRNA in the second transfection. BSR cells were transfected first with 6 capped ssRNAs (S1, S3, S4, S6, S8 & S9) and subsequently with all capped ssRNAs (column 1), all uncapped ssRNAs (column 2) or uncapped S9 together with remaining 9 capped ssRNAs (column 3). Each ssRNA was at 50 ng per transfection. The recovery of virus was shown as the total number of plaques per well (Mean ± SD).

Our previous study using a two-transfection reverse genetics (RG) system had suggested that the cap structure was not essential for genome packaging in BTV [50]. To further confirm the role of cap structure in BTV replication, we repeated the two-transfection RG schedule using for the first transfection only the genes that are essential for synthesis of the protein components of the primary replicase complex (S1, S3, S4, S6, S8, and S9) [50]. In the second transfection, the complete set of 10 ssRNA, all uncapped, were included as described in Materials and Methods. The lack of the capped RNA in all ten segments in the second transfection, which provides the BTV genome templates, reduced the efficiency of virus recovery (Fig. 5, column 2). However, lack of a cap structure on only the T7 S9 ssRNA did not reduce virus recovery (Fig. 5 column 3). It is noteworthy that the amount of VP4 synthesised by the first transfection of S4 into BSR cells was negligible and was incapable to form cap structure efficiently at 5′ end of an uncapped ssRNA [50]. These results indicated that, in addition to the initiation of translation and stabilization of mRNAs transcribed from core particles, there might be a role for cap structures for the enhancement of VP1 activity during assembly of the primary replication complex.

### Stimulation of polymerase activity by the 5′cap structure of template ssRNA is not due to priming or initial recognition of template but likely due to an allosteric modulation

**Figure 6:**
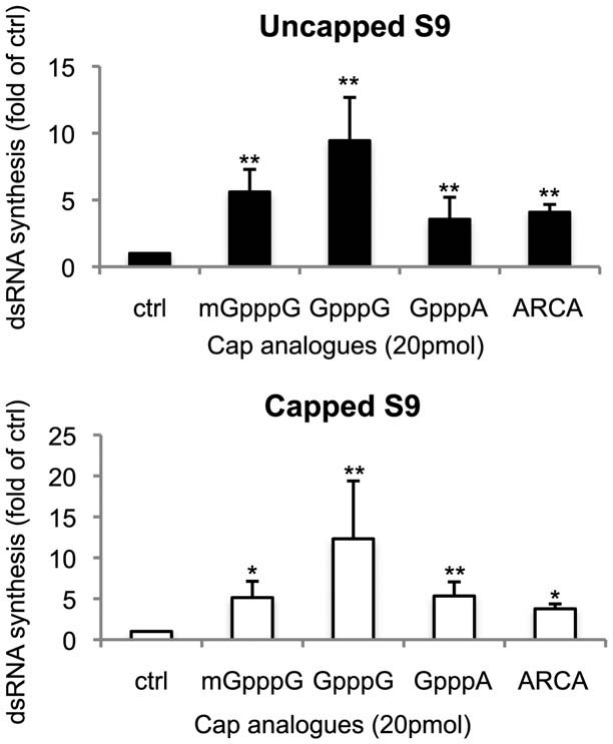
The effect of cap analogues on BTV polymerase activity. Each 20 pmol of cap analogues indicated at the bottom of each column, was added to the reaction mixture with 0.5 µg of uncapped (upper panel) or capped (lower panel) T7 S9 ssRNA and 70 µg of VP1. The radioactive intensity of each sample was standardized by control sample (ctrl), which was added water instead of cap analogue. The efficiency of dsRNA synthesis of each sample was shown as a fold of control (Mean ± SD). Asterisks are showing significant difference (**P<0.01; *P<0.05).

To further investigate the precise role of the cap structure in enhanced VP1 activity we made use of various cap analogues to determine their effect on dsRNA synthesis. Several types of cap analogue, 3′-O-methyl-m7GpppG (Anti-Reverse Cap Analogue, ARCA), m7GpppG, GpppG and GpppA were added to a standard reaction mixture containing 0.5 µg of capped or uncapped T7 S9 ssRNAs. Interestingly, the dsRNA synthesis from both capped and uncapped T7 S9 ssRNA was enhanced by the addition of all cap analogues, with GpppG showing the greatest enhancement (Fig. 6). Together with the kinetics data shown above, the fact that the presence of cap analogues did not compete for VP1 activity suggests that the 5′ cap structure of template ssRNA is not the primary element by which VP1 recognizes ssRNA template but that it acts to enhance the activity. Moreover, although there is the possibility that the enhancement of polymerase activity is due to artificial direct priming by the cap analogues, the fact that addition of the cap analogues enhanced the activity, together with the *in vivo* data shown above, suggests that the 5′ cap structure may act to stimulate activity *in trans*.

**Figure 7:**
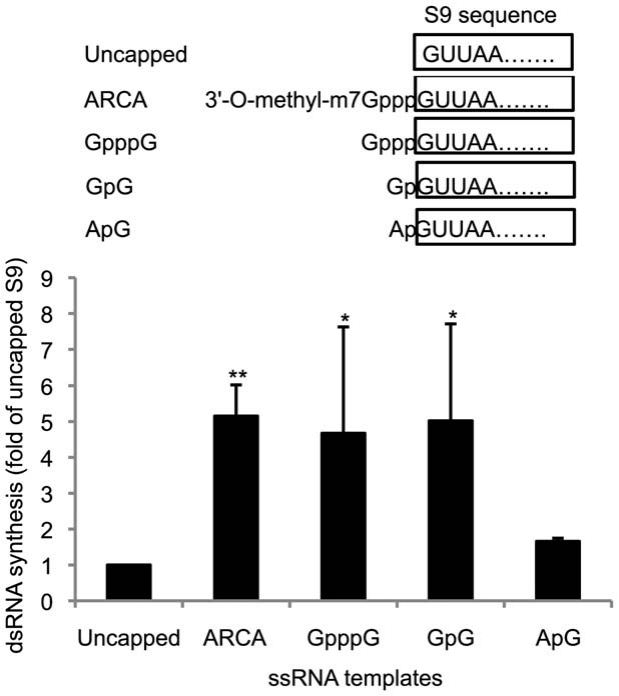
The effect of 5′ sequence of ssRNA template on BTV polymerase activity. Each 0.5 µg of uncapped (Uncapped, column 1), anti-reverse capped (ARCA, column 2), non-methylated capped (GpppG, column 3) or 5′ extended T7 S9 ssRNAs (GpG, column 4; ApG, column 5) was added to the reaction mixture. Schematic representation of each 5′-modified T7 S9 ssRNA was shown on the upper panel. The radioactive intensity of each sample was standardized by uncapped T7 S9 ssRNA sample (lower panel). The efficiency of dsRNA synthesis of each sample was shown as a fold of uncapped T7 S9 ssRNA sample (Mean ± SD). The radiolabeled bands were detected by autoradiography. The intensity of each band was counted using ImageJ software. Asterisks are showing significant difference (**P<0.01; *P<0.05).

T7 polymerase has a sequence preference, such as GpGpGp at the 5′ end of nucleotides [52]. To determine whether the enhancement of polymerase activity was due to sequence preference at the 5′ end of the template ssRNA, we modified the T7 S9 ssRNA by adding either guanosine (Gp-S9) or adenosine (Ap-S9) at the 5′ end of T7 S9 ssRNA and tested the efficiency of the dsRNA synthesis from these templates (Fig. 7, upper panel). In parallel, unmethylated capped T7 S9 ssRNAs (Gppp-S9) and methylated capped T7 S9 ssRNAs (3′-O-methyl-m7Gppp-S9) were tested for their effects on the dsRNA synthesis (Fig. 7, upper panel). The amount of dsRNA synthesized from each template was compared using quantitative autoradiography as described in Materials and Methods (Fig. 7, lower panel). The amount of dsRNA synthesized from Gp-S9 was five times more than that from uncapped T7 S9 ssRNA whereas the dsRNA synthesized from Ap-S9 did not increase (Fig. 7, columns 1, 4 and 5). Additionally, the dsRNA synthesis from unmethylated or methylated capped S9 ssRNA was higher than uncapped T7 S9 ssRNA (Fig. 7, columns 1–3). These results suggested that BTV VP1 has a sequence preference for GpG at the 5′ end, which may mimic the authentic cap structure, GpppG.

### Dinucleotides stimulate polymerase activity by priming the initiation of dsRNA synthesis

**Figure 8:**
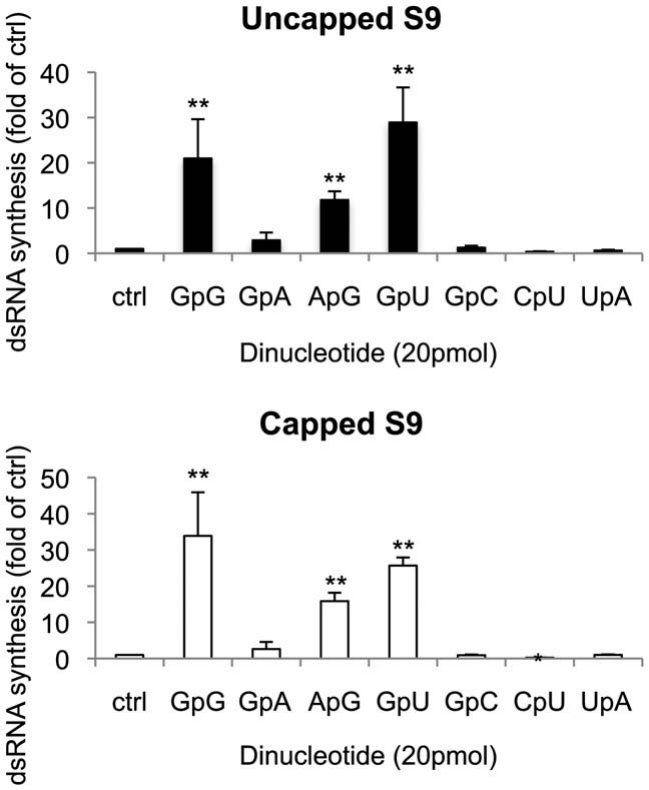
The effect of dinucleotides on BTV polymerase activity. Each 20 pmol of dinucleotide indicated at the bottom of each column was added to the reaction mixture containing 0.5 µg of either uncapped (upper panel) or capped (lower panel) T7 S9 ssRNA template. The radioactive intensity of each sample was standardized by control sample (ctrl, column 1), which was added water instead of dinucleotide. The efficiency of dsRNA synthesis of each sample was shown as a fold of control (Mean ± SD). Asterisks show significant difference (**P<0.01; *P<0.05).

**Figure 9:**
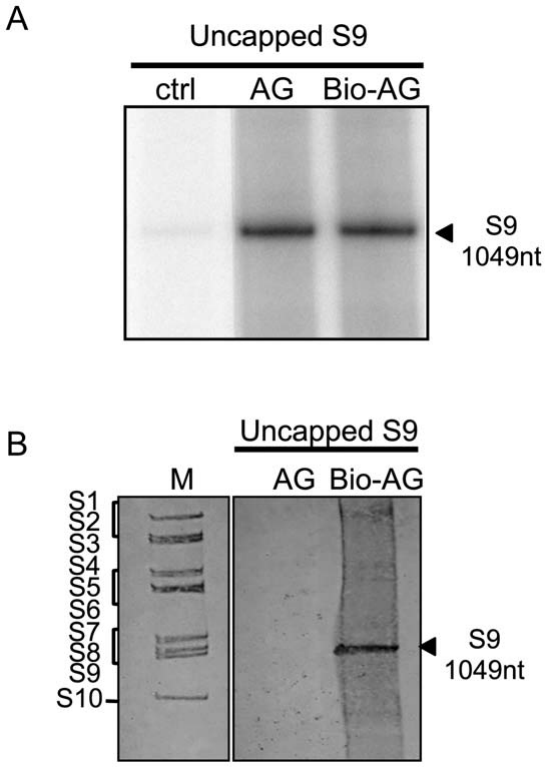
The priming activity of ApG. A. The effect of biotin-labeled ApG (Bio-ApG) on BTV polymerase was compared with non-labeled ApG (ApG). Each 20 pmol of ApG or Bio-ApG was added to the reaction mixture containing [α-^32^P] CTP. B. Priming activity by dinucleotide, ApG, was detected using biotin-labeled ApG and non-radioactive polymerase assay as described in Materials and Methods. As markers, BTV genomic segments was used and stained with 0.02% (w/v) methylene blue after transferring to the nylon membrane (lane 1). Non-labeled ApG (lane 2) or biotin-labeled ApG (lane 3) was added the reaction mixture containing non-labeled rNTP with uncapped T7 S9 ssRNA. Biotin-labeled dsRNAs were detected using Streptavidin*e-alkaline phosphatase* conjugate as described in Materials and Methods. Genome segments are indicated on the left.

Polymerase activities of some viruses, such as Influenza virus, Bovine viral diarrhea virus, Hepatitis C virus and GB virus-B, are initiated by dinucleotides due to priming the transcription initiation [41], [53], [54], [55]. Additionally, the study using rotavirus open cores showed that GpG, which complements the sequences of the 3′ end of rotavirus G8 ‘plus’ strand RNA, forms initiation complexes with VP1 and template ssRNA to initiate ‘minus’ strand synthesis [32]. To determine the effect of dinucleotide on the BTV polymerase activity, several types of dinucleotides were added to the polymerase reaction mixture. Three dinucleotides, GpG, GpA and ApG, which consisted of the same nucleotides as cap analogues, were tested with 0.5 µg of T7 S9 ssRNA. When the products were analysed the data showed clearly that GpG and ApG, which complement only the last nucleotide of the 3′ end sequence of BTV S9 plus strand RNA, strongly enhanced the polymerase activity (Fig. 8, columns 2 and 4). Similarly, biotinated pApG (biotin-ApG), which was incorporated only once at the 5′-end, also showed the strong enhancement of polymerase activity (Fig. 9A). In contrast, GpA did not enhance the observed activity (Fig. 8, column 3). Subsequently four more dinucleotides, GpU, GpC, CpU and UpA were tested for their effects on dsRNA synthesis. Of these, only GpU, which complements the last two bases of the 3′ end sequence of BTV S9 plus strand RNA, strongly enhanced the dsRNA synthesis whereas GpC, CpU and UpA, which fail to complement the 3′ end sequence of BTV S9 plus strand, did not exhibit any detectable enhancement (Fig. 8, columns 5–7). These results suggest that dinucleotides are capable of stimulating BTV polymerase activity by priming the initiation of dsRNA synthesis, similar to that observed for rotavirus VP1 [32].

To confirm this further, biotin-ApG was used for the non-radioactive polymerase assay as described in Materials and Methods. When biotin-ApG was added to the reaction mixture containing non-radiolabeled rNTPs, the newly synthesized dsRNAs were labeled with biotin at the 5′ end of the negative sense RNA and detected at the same position as S9 viral dsRNA stained with methylene blue (Fig. 9B). Interestingly, GpG always enhanced polymerase activity more than ApG. Additionally, the amount of dsRNA synthesized from Gp-S9 was at least five times more than that of uncapped T7 S9 ssRNA whereas synthesis of dsRNA from Ap-S9 did not increase (Fig. 7). Thus, while ApG could stimulate activity by priming, albeit less efficiently than GpU, GpG was superior, plausibly as a result of direct priming and allosteric stimulation, as it may mimic the effect of GpppG.

## Discussion

We previously demonstrated that BTV VP1 could act as a replicase in the absence of any other virus or host protein [23], [24], in contrast to rotavirus VP1, which failed to exhibit catalytic activity in the absence of the inner capsid protein VP2 [29]. In this study, we further confirmed the robustness and versatility of the BTV VP1 replicase activity by demonstrating that it could synthesize all ten dsRNAs simultaneously from BTV core-derived ssRNA templates in a single *in vitro* reaction in the absence of any other virus proteins. In addition, genomic dsRNA segments from rotavirus DLP-derived ssRNA templates that possess no sequence similarity with BTV also acted as templates, suggesting that this assay system could be an advantage for the future studies of *Reoviridae* RdRp.

The 3′ conserved sequence in rotavirus RNA segments is essential for polymerase activity [4], [29], [30], [32], [33]. In addition, although the initiation mechanism differs from the family *Reoviridae*, the 3′ end sequence is important for the pre-initiation events of bacteriophage phi6 RdRp, which assembles into a productive binary complex with template ssRNA [56]. However, in our preliminary study BTV VP1 did not require conserved termini for its catalytic activity, suggesting that BTV VP1 has sequence-independent replicase activity [24]. In the present study, we confirmed its sequence-independent activity by demonstrating that BTV VP1 could synthesize correct dsRNA segments from viral genomic templates of other *Reoviridae*. AHSV has 5′ and 3′ conserved regions in its genome similar to BTV genomic RNA whereas rotavirus genomes, especially the terminal conserved regions, are very different from BTV. Nonetheless, BTV VP1 could synthesize dsRNAs of rotavirus with correct sizes, suggesting strongly that BTV polymerase activity was sequence-independent *in vitro*. The amount of each synthesized segment was not equal. This phenomenon was also observed in rotavirus open core system [57]. There may be some structural constraints in certain ssRNAs. However, the dsRNA was well synthesized from single T7 ssRNA template of AHSV S4 unlike the AHSV core ssRNA template. Although further investigations are required, it may be that a mixture of several ssRNA segments in a single reaction may cause RNA-RNA interaction, thereby preventing the 3′ end of some ssRNA from reaching at the active site of VP1 and consequently resulting in uneven dsRNA synthesis. When non-viral ssRNAs were used as templates, BTV VP1 failed to synthesize dsRNAs of correct lane sizes. Smearing and many truncated bands were detected, suggesting premature termination as well as poor template recognition. This phenomenon is not due to the lack of cytidine at the 3′ end of non-vial ssRNAs as VP1 could still synthesize dsRNA from S2 mutant, which does not possess cystidine at 3′ end [24]. Several virus polymerase activities are already known to regulate their transcription by structure-based *cis*-acting replication elements in their genomic or subgenomic RNAs [58], [59], [60], [61], [62], [63]. In rotavirus replication, the presence of *cis*-acting functional elements of rotavirus ssRNAs has been suggested [30], [31], [64], [65], [66]. We previously demonstrated the presence of *cis*-acting sequences required for replication or packaging [50]. When the functional S9-EGFP transcript, S9E277/656, was added to the reaction, dsRNA synthesis was efficient, as expected. A role for a conserved feature in the templates of the family *Reoviridae*, required for polymerase activity, is suggested by these results although the precise nature and location remains to be determined.

The most important role of the 5′ cap structure in eukaryotic mRNAs is in the initiation of translation. However, it is also known to regulate RNA synthesis in virus replication [40], [43], [44], [45], [46]. The crystal structure of reovirus λ3 and rotavirus VP1 revealed a cap-binding site on the surface of their cage-like structures [3], [4], suggesting that the cap was the primary element by which VP1 interacts with and recognizes the 5′ end of a positive-strand. The putative model structure of BTV VP1 has a strong similarity with the RdRp structures of other members of the family [26] and our data here suggest that this similarity extends to cap recognition by, and stimulation of, replicase activity in BTV. As demonstrated by the competition assays with various cap analogues and kinetic analysis, this effect was not due to direct VP1 recognition of the 5′ end of the ssRNA template that is suggested for other members of the family [3], [4], but it is likely due to an allosteric modulation. Precise kinetic support for the effect of cap structure for RdRps activity may come from further investigation of BTV VP1 and other members of the RdRp family. Our data also demonstrate that the cap structure is unlikely to play any role in genome packaging of BTV, although it could be important for dsRNA synthesis during primary replication. The influence of the cap structure in virus replication is worthy of future investigation using additional reverse genetics experiments.

A dinucleotide, GpG, had been shown to be incorporated into the 5′-end of the newly synthesized negative sense RNA, suggesting that GpG primes dsRNA synthesis by forming the replication initiation complex with template RNA and RdRp in the early step of rotavirus replication [32]. Our results also showed that dinucleotides GpG, ApG and GpU could stimulate BTV VP1 replicase activity by priming although priming by either GpG or ApG resulted in synthesis of artificial dsRNA with the 5′-overhang of negative sense RNA. This indicated that the last nucleotide, ‘G’ of the dinucleotides that complemented the 3′ end of the template sequence was sufficient to prime the activity. This feature of BTV VP1 is not shared by rotavirus VP1 as ApG failed to prime in rotavirus open core polymerase assay [32]. There are several characteristics of BTV VP1 activity that are noteworthy. For example, dsRNA synthesis from Gp-S9 ssRNA template was more efficient than that from uncapped S9 ssRNA template and the GpG was a better stimulator for polymerase activity than that of ApG. This indicates that in addition to priming, GpG could enhance the activity by allosteric effect, possibly due to some similarity to GpppG. Although further study is necessary to clarify the effect of GpG and ApG, and in addition if they possess allosteric effect, our results indicated that the replicase activity of BTV VP1 could be stimulated by dinucleotide priming at the initiation of replication.

In summary, in this study we showed two stimulation factors of VP1 replicase activity, allosteric effect of cap structure and priming effect of dinucleotides as well as the possible presence of *cis*-acting element shared by ssRNAs in the members of family *Reoviridae*. Our system for polymerase assay could be modified for *in vitro* assembly assay of the replication complex to further clarify the mechanism of BTV replication in the future.

## Materials and Methods

### Expression and Purification of his-tagged BTV VP1

The his-tagged VP1 was expressed in the *Spodoptera frugiperda* (*Sf9*) cell line (ATCC, Rockville, MD) and purified as described previously with some modifications [24]. Briefly, *Sf*9 cells were infected with AcBTV10.NHis1 at a multiplicity of infection of 5.0. At 2 days post-infection, cells were harvested and lysed with VP1 lysis buffer (50 mM sodium phosphate pH8.0, 10% (v/v) glycerol, 0.5% (v/v) Nonidet P-40) containing 1×protease inhibitors (Protease Inhibitor Cocktail Set V EDTA-Free, Calbiochem). Nuclei and cell debris were removed by centrifugation at 9400 *g* for 1 hour (h) at 4°C. The cell lysate was mixed with HIS-Select® Nickel Affinity Gel (Sigma) for 1 h at 4°C. After washing the affinity gel with 50 mM sodium phosphate buffer (pH 8.0) containing 10% glycerol and 20 mM imidazole, his-tagged VP1 was eluted with 50 mM sodium phosphate buffer (pH 8.0) containing 10% glycerol and 300 mM imidazole. The eluted his-tagged VP1 was diluted at one in five with 50 mM Tris-HCl buffer (pH 8.0) containing 10% glycerol and 1 mM DTT and further purified by the affinity column, Hi-Trap® Heparin HP column (GE Healthcare), using AKTA system (GE Healthcare) with a linear sodium chloride gradient (100 mM-1000 mM).

### Preparation of single-stranded RNA (ssRNA) for polymerase assay and use in reverse genetics recovery of virus

Single-stranded RNAs (ssRNA) of BTV, African horse sickness virus (AHSV), simian rotavirus, rhesus rotavirus and bovine rotavirus were synthesized *in vitro* using purified core particles or double-layered particles of each virus (core ssRNA or DLP ssRNA) as described previously [51], [67], [68], [69]. For synthesis of ssRNA with T7 RNA polymerase (T7 ssRNA), T7 plasmids constructed previously [49], [50] were used. All BTV genome sequences were deposited in GenBank (Accession numbers: FJ969719, FJ969720, FJ969721, FJ969722, FJ969723, FJ969724, FJ969725, FJ969726, NC006008, NC006015, FJ969727 and FJ969728). For synthesis of 5′ extended S9 ssRNAs, EGFP ssRNA and puromycin N-acethyltransferase (PAC, puromycin resistant gene) ssRNA, PCR products were used as templates. The following primers were used for the PCR amplification: T7EGFP-F (5′- taatacgactcactatagggATGGTGAGCAAGGGCGAGGA), T7PUR-F (taatacgactcactatagggATGACCGAGTACAAGCCCAC), T7GS9-F (5′-taatacgactcactataGGTTAAAAAATCGCATATGTC), T7AS9-F (5′-taatacgactcactataAGTTAAAAAATCGCATATGTC), EGFP-R (5′-tttccatggTTACTTGTACAGCTCGTCCA), PUR-R (5′-ctaagatctTCAGGCACCGGGCTTGCGGG) and S9-R (5′- GTAAGTGTGAAATCGCCCTACGTCA). For synthesis of luciferase ssRNA, control linear DNA from RiboMAX Large Scale RNA Production System-T7 kit (Promega) was used. Capped T7 ssRNAs were synthesized using mMESSAGE mMACHINE T7 Ultra Kit (Ambion) according to manufacturer's protocols. For synthesis of uncapped T7 ssRNAs and unmethylated capped T7 ssRNA, RiboMAX Large Scale RNA Production System-T7 (Promega) was used according to the manufacturer's procedures. The unincorporated nucleotides and cap analogues were removed using Illustra MicroSpin G25 columns (GE Healthcare) during standard phenol/chloroform purification methods. The synthesized ssRNAs were dissolved in nuclease-free water, and stored at −80°C.

### Polymerase assay

Polymerase assay was performed as described previously with some modifications [24]. Briefly, 70 ng of his-tagged VP1 and several amounts of ssRNA templates were added to 50 µl of reaction mixture (50 mM Tris-HCl pH7.4, 6 mM magnesium acetate, 600 µM Manganese chloride, 320 µM ATP, 320 µM GTP, 320 µM UTP, 2 µM CTP, 0.2 µCi/µl [α-^32^P] CTP (Perkin Elmer), 2% (w/v) PEG4000, and 1.6 U RNasin plus (Promega) in presence and absence of 20 pmol of cap analogues (New England Biolabs) or 20 pmol 5′-hydroxyl dinucleotides (IBA). Note that ssRNA templates were not denatured. After incubation for 5 h at 37°C, synthesized dsRNAs were purified using a standard phenol/chloroform method and analyzed using native-PAGE. Note that the dsRNA synthesis in the VP1 reactions proceeded in a linear manner for at least 5 h [24]. As markers, end-labeled- dsRNA genomes purified from virus-infected cells were used as described previously [24]. Gels were dried and exposed to Storage Phospher screen (GE HEalthcare). The radiolabeled dsRNAs were detected using the image analyzer, Typhoon Trio (GE HEalthcare), and each radiolabeled band was quantified using ImageJ software (NIH: http://rsb.info.nih.gov/ij/).

The experimental data was then fitted by a nonlinear regression method using the program Prism (GraphPad Software, USA). The kinetics parameters were determined by the Michaelis-Menten equation:
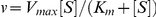
where [*S*] is the substrate concentration (ng/µl); *K_m_* is the apparent Michaelis-Menten constant; and *V_max_* is the maximal rate attained when the enzyme active sites are saturated by substrate (quantified radioactive count/min).

### Transfection of cells with BTV transcripts

BSR monolayers in twelve-well plates were transfected twice with BTV mRNAs using Lipofectamin 2000 Reagent (Invitrogen) as describe previously [50]. BTV transcripts were mixed in Opti-MEM (Invitrogen) containing 0.5 U/µl of RNasin plus (Promega) before being mixed with Lipofectamin 2000 Reagent in Opti-MEM containing 0.5 U/µl of RNasin plus. The transfection mixture was incubated at 20°C for 20 min and then added directly to cells. The first transfection was performed with a standard of 50 ng of each T7 transcript (S1, S3, S4, S6, S8 and S9), and a second transfection, again with 50 ng of each of the ten T7 transcripts, at 18 h post first transfection. At 6 h post second transfection, the culture medium was replaced with 1.5 ml overlay consisting of DMEM, 2% FBS, and 1.5% (wt/vol) agarose type VII (Sigma) and the plates were incubated at 35°C in 5% CO_2_ for 3 days to allow plaques to appear.

### Priming assay

A modified method of polymerase assay was used for measuring dinucleotide priming. Briefly, 70 ng of His-tagged VP1 and 0.5 µg of T7 S9 ssRNA were added to 50 µl of reaction mixture (50 mM Tris-HCl pH7.4, 6 mM magnesium acetate, 600 µM Manganese chloride, 320 µM ATP, 320 µM GTP, 320 µM UTP, 320 µM CTP, 2% (w/v) PEG4000, and 1.6 U RNasin plus (Promega)) in presence of 20 pmol of biotin-labeled ApG (IBA). After separation by native-PAGE gel, samples were transferred to the positive-charged nylon membrane, Hybond N+ (GE Healthcare) and Biotin-labeled dsRNA bands were detected using Streptavidine-*alkaline phosphatase c*onjugate (Sigma) and BCIP®/NBT Alkaline Phosphatase Substrate (Sigma) by the same method as with the Biotin Luminescent Detection Kit (Roche Applied Science). As markers, purified dsRNA genomes were used and stained with methylene blue solution (0.02% (w/v) methylene blue, 0.3 M sodium acetate, pH 5.0) after transferring to the nylon membrane.
